# Clinical Improvement and Taxonomic–Functional Gut Microbiome Remodeling After Six Months of Multi-Strain Synbiotic Supplementation in Mexican Children with Autism Spectrum Disorder

**DOI:** 10.3390/nu18152441

**Published:** 2026-07-26

**Authors:** Amapola De Sales-Millan, Paulina Reyes-Ferreira, Rina María González-Cervantes, Mariana Luna-Álvarez, Sara Guillén-López, José F. Cobo-Díaz, Sandra Ramos, José Félix Aguirre-Garrido, José Antonio Velázquez-Aragón

**Affiliations:** 1 Doctorado en Ciencias Biológicas y de la Salud, Universidad Autónoma Metropolitana, Ciudad de Mexico 04960, Mexico; 2212803115@correo.ler.uam.mx; 2 Departamento de Salud Mental, Instituto Nacional de Pediatría, Ciudad de Mexico 04530, Mexico; paulinareyesferreira@gmail.com; 3 Departamento de Ciencias Ambientales, Universidad Autónoma Metropolitana-Lerma, Lerma 52006, Estado de Mexico, Mexico; rgonzalez@correo.ler.uam.mx; 4 Grupo Neurológico, Neuroquirúrgico y de Columna, Hospital Ángeles Acoxpa, Ciudad de Mexico 14308, Mexico; mariana_luna93@hotmail.com; 5 Laboratorio de Errores Innatos del Metabolismo y Tamiz, Instituto Nacional de Pediatría, Ciudad de Mexico 04530, Mexico; sguillenl@pediatria.gob.mx; 6 Departamento de Higiene y Tecnología de los Alimentos, Facultad de Veterinaria, Universidad de León, 24071 Leon, Spain; 7 Laboratorio de Oncología Experimental, Instituto Nacional de Pediatría, Ciudad de Mexico 04530, Mexico; sramosa@pediatria.gob.mx

**Keywords:** autism spectrum disorder, probiotics, synbiotics, gut microbiota, microbiome function, ASD severity, gastrointestinal symptoms

## Abstract

**Background/Objectives**: Gut dysbiosis in children with autism spectrum disorder (ASD) has been associated with alterations in microbial ecology and metabolic function that may contribute to gastrointestinal dysfunction and the severity of clinical manifestations. Synbiotic and probiotic supplementation has emerged as a promising microbiome-targeted strategy for ASD; however, its effects on gut microbiome composition, functional potential, and clinical outcomes remain incompletely understood. We conducted a longitudinal study of Mexican children diagnosed with ASD to analyze changes in the composition, diversity, and functional potential of the gut microbiome during six months of multi-strain synbiotic supplementation. **Methods**: Stool samples were collected from 25 children with ASD at baseline and after 3 and 6 months of multi-strain synbiotic supplementation. Gut microbiome composition and diversity were analyzed by 16S rRNA gene sequencing, whereas whole metagenome sequencing (WMS) was performed in a subset of samples to evaluate the functional potential of the fecal microbiome. Gastrointestinal symptoms were assessed using the Rome IV criteria, and ASD severity was evaluated with the Childhood Autism Rating Scale (CARS). **Results**: Twenty-five children with ASD completed the 6 months of synbiotic supplementation. Overall, ASD severity decreased, reflected by a reduction in total CARS score, and improvements in several CARS domains. Gastrointestinal symptoms also decreased significantly. Longitudinal microbiome profiling revealed significant taxonomic and diversity changes over the supplementation period, while WMS identified changes in microbial metabolic potential, including enrichment of tryptophan biosynthesis pathways and reduced L-rhamnose degradation. **Conclusions**: This exploratory research provides proof-of-concept evidence supporting multi-strain synbiotic supplementation in children with ASD. Larger controlled studies are needed to confirm these findings and clarify their relevance to microbiota–gut–brain axis interactions. The observed concordance between clinical improvements and microbiome remodeling supports further investigation of microbiome-targeted interventions according to ASD severity and duration of supplementation.

## 1. Introduction

Autism spectrum disorder (ASD) is a complex neurodevelopmental condition characterized by impairments in social interaction and communication, as well as restricted and repetitive patterns of behavior and stereotyped movements, with clinical presentations ranging from mild to severe [1]. The prevalence of ASD has increased in recent decades, exceeding 2% in some populations and being almost four times higher in males than in females [2,3]. Although the etiology of ASD is multifactorial and involves both genetic and environmental factors [4], gut microbiota have attracted increasing attention because they represent a dynamic and potentially modifiable biological system associated with gastrointestinal dysfunction, immune regulation, microbial metabolite production, and gut–brain axis signaling [5,6].

Gastrointestinal symptoms are frequent in children with ASD, with reported prevalence ranging from 20% to 70%, and have been associated with greater severity of behavioral manifestations, including sensory processing difficulties, sleep disturbances, communication and language problems, anxiety, irritability, and social withdrawal [7,8,9]. However, these associations should not be interpreted as evidence that gut dysbiosis directly causes or worsens ASD symptoms. Rather, current evidence suggests that alterations in microbial ecology may contribute to, or reflect, biological processes involved in gastrointestinal dysfunction and gut–brain axis communication. Therefore, gut microbiota are central to the present study, not as an isolated causal factor, but as a modifiable biological interface potentially linking gastrointestinal, metabolic, immune, and neurobehavioral features in ASD.

Several studies have reported differences in the gut microbiota of children with ASD compared with neurotypical children (NT), including reduced abundance of short-chain fatty acid-producing bacteria, enrichment of potentially pro-inflammatory taxa, and alterations in microbial metabolic pathways involved in immune regulation, neurotransmitter metabolism, and gut–brain axis signaling [10]. In line with this evidence, our previous work identified both taxonomic and functional differences in the gut microbiota of children with ASD compared with NT children [11]. These findings support the relevance of microbiome-targeted interventions, such as prebiotics, probiotics, and synbiotics, aimed at modulating microbial ecology and function rather than simply replacing specific bacterial taxa.

From an ecological perspective, a healthy gut microbiome is characterized by resilience, defined as the ability to resist perturbations and recover its ecological structure and functional activity while maintaining ecosystem stability [12]. This concept provides a rationale for microbiome-targeted interventions aimed at promoting microbial homeostasis. Because different probiotic strains may exert distinct metabolic, immunomodulatory, and intestinal barrier-supporting functions, multi-strain synbiotic formulations have been proposed to provide complementary ecological effects [13]. Although direct comparisons with single-strain interventions remain limited, this approach may promote broader modulation of gut microbial function and gut–brain axis signaling.

Previous intervention studies in children with ASD have reported heterogeneous findings. A recent study showed that six months of *Lactiplantibacillus reuteri* supplementation did not significantly alter overall microbiome composition but was associated with improved social behavior in children with ASD [14]. Similarly, supplementation with *Lactiplantibacillus plantarum* PS128 for 28 days has been reported to alleviate oppositional and defiant behaviors and symptoms of attention deficit hyperactivity disorder [15]. However, systematic reviews and meta-analyses of randomized controlled trials found significant improvements in gastrointestinal symptoms but did not confirm a positive effect of prebiotics and probiotics on ASD symptoms [16,17,18]. These findings highlight the need for additional studies that integrate clinical outcomes with taxonomic and functional analyses of the gut microbiome, particularly in longitudinal designs.

Given the limited evidence integrating longitudinal clinical follow-up with gut microbiome compositional and functional analyses in children with ASD, the objective of this study was to evaluate whether six months of multi-strain synbiotic supplementation was associated with changes in gut microbiome composition, diversity, and functional potential, and whether these changes were accompanied by modifications in gastrointestinal symptoms and ASD severity.

## 2. Materials and Methods

### 2.1. Participants, Study Design and Ethic Approval

Children of both sexes aged 4–11 years were eligible for participation. Children with syndromic comorbidities or those who did not complete the six-month synbiotic intervention were excluded. Participants who had used probiotics, prebiotics, or oral antibiotics during the six months preceding enrollment were also excluded to minimize potential effects on gut microbiome composition. Data were collected between May 2022 and November 2023. This longitudinal study evaluated the nutritional status, severity of ASD, gastrointestinal symptoms, composition, diversity, and functional potential of the gut microbiome of children with ASD at a 6-month follow-up. During the 6-month period (evaluated three times; before: T0, after 3 months: T3, and after 6 months of supplementation: T6), the patients consumed synbiotics, and no dietary intervention was implemented.

This longitudinal study is part of a research protocol with registration number INP 2021/058, approved on October 8^th^, 2021, by the Research Ethics Committee of Instituto Nacional de Pediatría (INP) Mexico and conducted in accordance with the Declaration of Helsinki. The participants were recruited via convenience sampling from the Mental Health service of INP in Mexico City. All ASD clinical assessments, including the Autism Spectrum Disorder Diagnostic Interview (CRIDI-ASD) and Childhood Autism Rating Scale (CARS) were performed by the same clinical psychologist, who was experienced in the administration of these standardized instruments. Using a single evaluator ensured consistency across assessments and minimized inter-rater variability. All participants received oral and written information about the study, and written consent was obtained, ensuring the confidentiality of personal information.

### 2.2. Synbiotic Supplementation

A commercial multi-strain synbiotic manufactured by Italmex S.A, Mexico City, Mexico, was used in this study and was provided as a dry powder containing: *Lacticaseibacillus acidophilus* (1 billion CFU), *Lacticaseibacillus casei* (1 billion CFU), *Lacticaseibacillus rhamnosus* (440 million CFU), *Lactiplantibacillus plantarum* (176 million CFU), *Bifidobacterium infantis* (27.6 million CFU) and *Streptococcus thermophilus* (667,000 CFU) + inulin enriched with oligofructose (50 mg) per 1 g. The participants’ caregivers were instructed to give them one sachet of synbiotic once a day with water after meals. The synbiotic intervention began after baseline samples (T0) were collected and lasted six months. Adherence to the synbiotic regimen was measured using an adherence calendar completed by the caregivers. The principal investigator closely monitored for any potential side effects.

### 2.3. Anthropometric Measures

Two trained dietitians obtained duplicate anthropometric measurements following WHO recommendations [19]. Height was measured using a SECA 206 stadiometer, and weight was measured using a digital pediatric Seca^®^ scale (SECA 206, Hamburg, Germany), following the anthropometric methodology recommended by the WHO. Weight and height measurements were used to calculate the body mass index (BMI) expressed in kg/m^2^. The WHO Anthro Plus software, v.1.0.4, was used to determine the BMI for age Z-score (BMI/A). Nutritional diagnoses were evaluated according to the WHO 2007 [19] standards, which consider underweight as <−2 SD, normal weight −2 to 1 SD, overweight > 1 SD, and obesity > 2 SD.

### 2.4. Assessment of ASD Severity and Gastrointestinal Symptoms

CARS was used to determine ASD severity before supplementation (T0) and after 6 months of supplementation (T6), as changes in core behavioral symptoms are expected to occur gradually and are more appropriately evaluated after a longer intervention period.

To detect gastrointestinal symptoms, the Rome IV Criteria questionnaire (school-age children section) was administered by a single specialist physician using a standardized interview format. Because the participants were children, the questionnaire was completed based on information provided by their parents or primary caregivers, who were asked about the presence of gastrointestinal symptoms in each patient. Gastrointestinal symptoms were assessed at baseline (T0), after 3 months (T3), and after 6 months (T6) to monitor the temporal evolution of gastrointestinal responses during the intervention, as these symptoms may change earlier than behavioral manifestations.

### 2.5. Fecal Sample Collection, DNA Extraction and Sequencing

After being provided with standardized collection instructions, participants’ caregivers collected approximately 1–5 g of freshly passed stool into sterile containers. Upon receipt, the stool samples were refrigerated at 4 °C to preserve their integrity during transport. Within 4 h, the samples were delivered to the laboratory, where aliquots of approximately 200 milligrams each were individually extracted and frozen at −70 °C until further processing. Samples were collected at three time points: T0 (n = 25), T3 (n = 25), and T6 (n = 25), with a total of 75 stool samples.

DNA extraction was performed with QIAamp^®^ PowerFecal^®^ Pro DNA kit (cat. 51804-50, QIAGEN^®^, Redwood, CA, USA) following manufacturer protocol. The quantification of purified fecal DNA was performed by evaluating its absorbance at 260 nm, and its purity was determined by calculating the absorbance ratios at 260/280 and 260/230 nm using a NanoDrop Lite spectrophotometer (Thermo Scientific, Waltham, MA, USA). The integrity of DNA was assessed by electrophoresis on 1.0% agarose gels.

The V3–V4 region of the 16S rRNA gene was amplified using the primers 341F (CCTACGGGNGGCWGCAG) and 805R (GACTACHVGGGTATCTAATCC). The sequencing was performed using an Illumina MiSeq platform and 300 bp paired-end sequencing. For each sample, 150,000 raw reads with an average length of 464 bp were generated according to the Integrated Microbiome Resource (IMR) of Dalhousie University, Canada (https://imr.bio/pricing.html (accessed on 15, February, 2024)). Additionally, whole metagenome sequencing (WMS) was performed for functional analysis on 40 samples of 20 children that showed changes greater than 5 points between T0 and T6 in the total CARS score. In this case, DNA was sequenced on an Illumina NextSeq 2000 platform following the IMR of Dalhousie University, Canada (https://imr.bio/pricing.html (accessed on 29, May, 2024)), to generate at least 10 Mreads per sample by 150 paired-end sequencing. Sequences were submitted to the National Center for Biotechnology Information (NCBI) under the BioProject accession number PRJNA1425957.

### 2.6. Bioinformatic and Statistical Analysis

Raw reads of the V3–V4 region from 16S rRNA were analyzed using Divisive Amplicon Denoising Algorithm 2 (DADA2) v.3.9 R package (https://benjjneb.github.io/dada2/download.html (accessed on 08, July, 2024)). The taxonomy assignment was performed by aligning the sequence of each Amplicon Sequence Variant (ASV) to the Silva v.138 database (https://benjjneb.github.io/dada2/training.html (accessed on 08, July, 2024)). Rarefaction curves were done by plotting the number of sequences versus the number of ASVs to assure that sequencing depth was sufficient to capture most of the prokaryotic richness in each sample. Alpha diversity was reported in terms of Richness, Shannon index and Simpson index using the “vegan” R package (v2.6.6.1). The β-diversity was estimated by Principal Coordinates Analysis (PCoA) using Bray–Curtis dissimilarity, and the Adonis permutational test was used to evaluate overall differences in bacterial community structure between the k-mean-categorized groups with the “vegan” R package. A canonical correspondence analysis (CCA) was performed using the main bacterial genera to explore associations between severity level and supplementation duration with the microbiota in relation to the covariates, using the Paleontological Statistics (PAST) software v.3.0.

For WMS analysis, BioBakery v3 pipeline [20] was used to process the raw reads. Trimgalore (v0.6.1-5) (https://github.com/FelixKrueger/TrimGalore (accessed on 27, August, 2024)) was used to trim adapters from raw sequence reads and remove reads matching a human genome reference. Metagenomic Phylogenetic Analysis (MetaPhlAn v4.1.1) [21,22] was used to generate taxonomic profiles by aligning reads to a reference database of marker genes mpa_vJun23_CHOCOPhlAnSGB_202403 (http://cmprod1.cibio.unitn.it/biobakery4/metaphlan_databases/ (accessed on 28, August, 2024)). Finally, metabolic pathways and gene families were determined using the Human Microbiome Project Unified Metabolic Analysis Network (HUMAnN v3.9) [20] with the MetaCyc (http://metacyc.org/ (accessed on 28, August, 2024)) and UniRef90 diamond full reference database (version 201901b) (https://github.com/biobakery/humann#custom-protein-reference-database (accessed on 28, August, 2024)).

For statistical analysis, absolute frequencies and percentages were determined for qualitative variables, and quantitative variables were expressed as means and standard deviations (SDs). A paired *t*-test for related samples was used to compare changes between supplementation times with microbiota, functionality and covariates, in the statistical software SPSS version 22 with a *p*-value < 0.05. Abundance, alpha and beta bacterial diversity statistical analyses were performed with software R version 3.6.0, and figures were drawn with the “ggplot2” R package (v3.5.1). Comparisons between multiple groups of samples for taxonomy were performed by using the Kruskal–Wallis test and the post hoc Wilcoxon signed-rank test. *p*-values were adjusted through the Benjamini & Hochberg method for multiple comparisons and significance was established at *p* < 0.05. To identify changes in the ASD Severity and Supplementation Time along the study, a repeated measures two-tailed Fisher’s exact test was performed. Furthermore, the Spearman correlation was used to analyze the correlation between differentially abundant taxa and other variables as abundance of metabolic pathways. These tests were performed with the GraphPad Prism analysis package, version 9.5.1.

## 3. Results

### 3.1. Demographic, Anthropometric, Nutritional Status, ASD Severity, and Gastrointestinal Symptoms of the Participants

**Figure 1:**
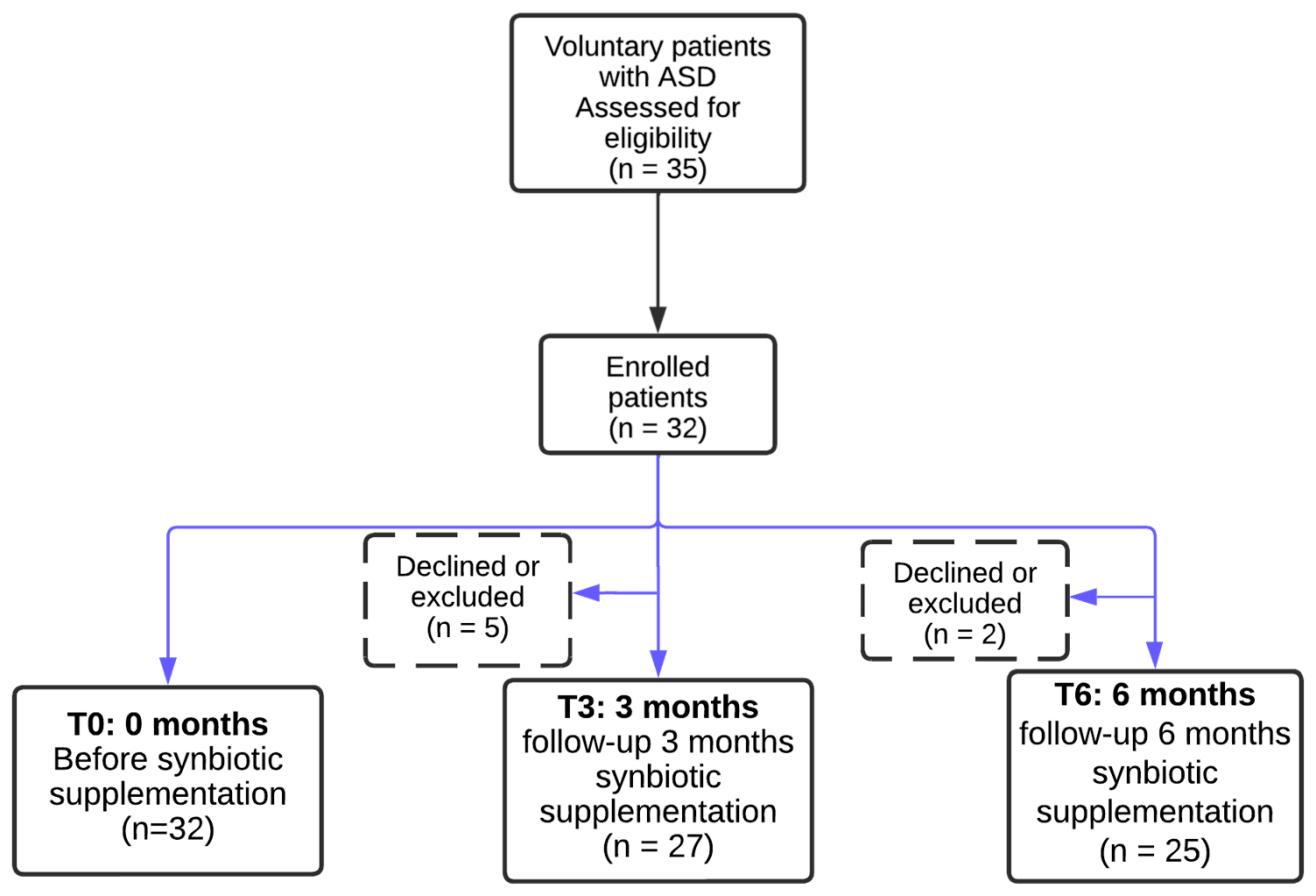
Flow diagram of participant recruitment, follow-up, and gut microbiota analysis in fecal samples across the study.

A total of 35 patients diagnosed with ASD were recruited for the study, of whom 25 completed the 6-month follow-up of symbiotic supplementation (Figure 1). Adherence to treatment was >85% in all participants and had a mean value of 90.88%. Table 1 shows the characteristics of the study participants.

**Figure 2:**
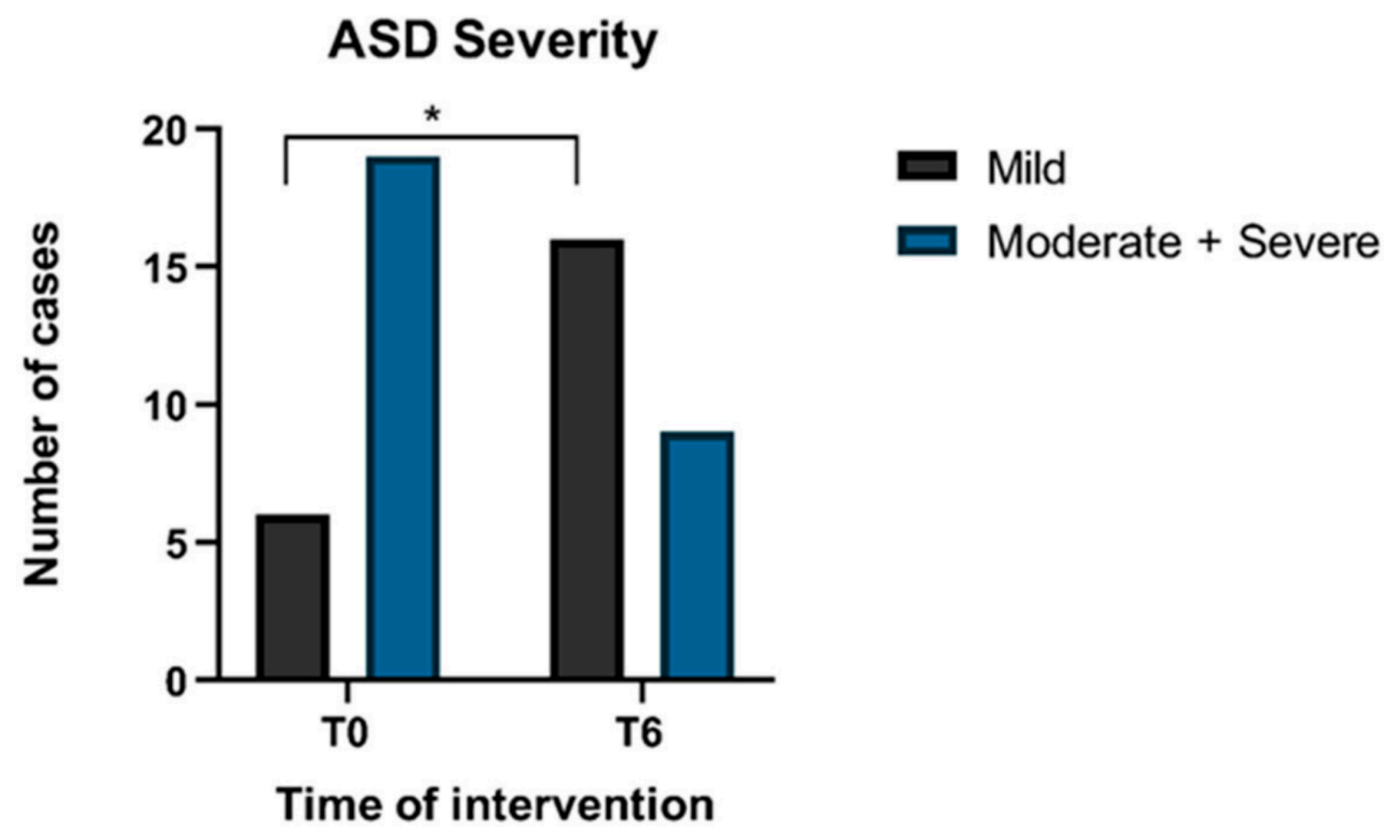
Differences in ASD severity after synbiotic supplementation. Black bar corresponds to mild severity and blue to moderate + severe severity. * Two-tailed Fisher’s exact test *p* < 0.05.

The mean age before synbiotic supplementation was 6.72 ± 2.03 years, and at six months the mean age was 7.48 ± 2.02 years. The sample consisted predominantly of males (84%). According to the BMI Z-score, the percentage of individuals with normal weight was 36% before the start of synbiotic supplementation, and during the 6-month follow-up, the percentage of individuals with normal weight changed to 56%; an increase in obesity (16–28%) and a decrease in underweight (24–4%) were noted, although these changes were not statistically significant (*p* > 0.05). ASD severity was mostly moderate (40%) before synbiotic supplementation and mostly mild (64%) at the 6-month follow-up. A statistically significant change (*p* < 0.009) was observed when comparing the number of individuals in the group with mild manifestations (mild) and the group with moderate or severe manifestations (moderate + severe) at baseline and six months after the intervention (Figure 2). The most frequent gastrointestinal symptoms before supplementation were flatulence (76%), irritable bowel syndrome (48%), and reflux (40%), followed by vomiting (36%), abdominal pain (36%), and constipation (32%); these same symptoms decreased significantly (*p* < 0.05) after 6 months of supplementation.

### 3.2. Changes in the Childhood Autism Scale During Synbiotic Supplementation Follow-Up

CARS scores decreased significantly in several domains after six months of synbiotic supplementation, including emotional response, body use, object use, adaptation to change, auditory response, fear or nervousness, and general impression (Table 2). The total CARS score also decreased significantly from T0 to T6 (*p* = 0.007).

### 3.3. Changes in Relative Microbial Abundance at Different Taxonomic Levels During Synbiotic Supplementation

**Figure 3:**
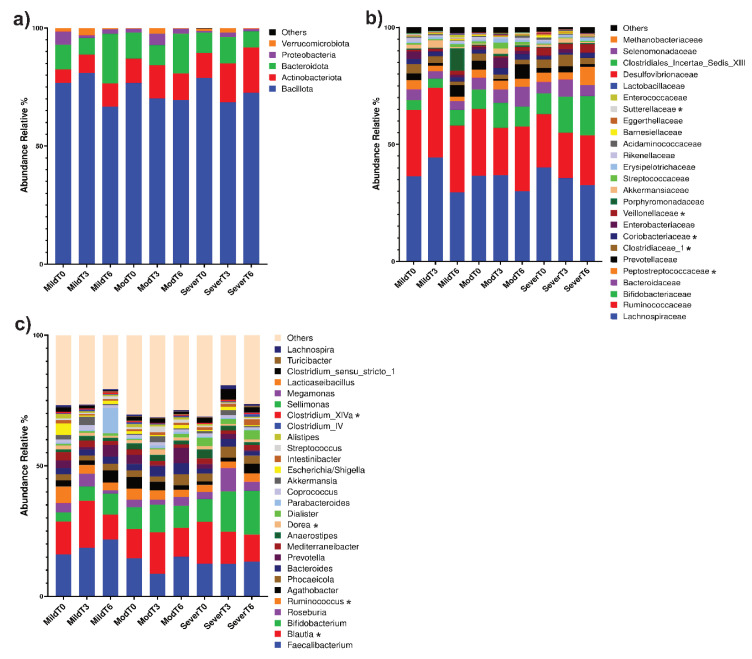
Relative abundance of the gut microbiota at (**a**) phylum, (**b**) family, and (**c**) genus taxonomic levels according to ASD severity and supplementation (mild, moderate and severe) time (T0, T3 and T6). Statistically significant differences (*p* < 0.05) are indicated by asterisk at the legends.

The gut microbiome composition analysis through sequencing of the 16S rRNA gene of children with ASD according to severity (mild: n = 6; moderate: n = 12 and severe: n = 7) and supplementation time (T0, T3 and T6) is shown in Figure 3a–c. Changes with statistical significance (*p* < 0.05) were observed at the family level: *Clostridiaceae_1* ModerateT0 vs. ModerateT6, *Veillonellaceae* ModerateT3 vs. ModerateT6 and ModerateT0 vs. ModerateT6, *Sutterellaceae* ModerateT3 vs. ModerateT6; *Peptostreptococcaceae* SevereT3 vs. SevereT6 and SevereT0 vs. SevereT6, *Coriobacteriaceae* SevereT0 vs. SevereT3; *Clostridium_XIVa* ModerateT3 vs. ModerateT6 in the structure of microbial communities according to the relative abundance of bacterial populations in patient samples when comparing supplementation times and the degree of severity of ASD.

Analysis comparing the relative abundance between supplementation time showed significant differences in some taxa at different taxonomic levels (phylum to genus with 16S rRNA gene) (Table 3), with the following results: at the phylum level, there were significant differences in Bacillota (T0 vs. T6) and Verrucomicrobia (T3 vs. T6); at the class level, there were significant differences in *Clostridia* (T0 vs. T6), *Verrucomicrobiae* (T3 vs. T6), and *Betaproteobacteria* (T3 vs. T6); at the order level there were significant changes in *Clostridiales* (T0 vs. T6), *Veillonellales* (T0 vs. T6), *Verrucomicrobiales* (T3 vs. T6) and *Burkholderiales* (T3 vs. T6); at the family level, there were significant changes in *Lachnospiraceae* (T3 vs. T6) and (T0 vs. T6), *Clostridiaceae_1* (T0 vs. T6), *Veillonellaceae* (T0 vs. T6) and *Akkermansiaceae* (T3 vs. T6); at the genus level, there were significant differences in *Blautia* (T3 vs. T6), *Mediterraneibacter* (T0 vs. T6), *Anaerostipes* (T3 vs. T6), *Akkermansia* (T3 vs. T6), *Clostridium_XlVa* (T3 vs. T6), *Sellimonas* (T0 vs. T3) and (T0 vs. T6) and *Lachnospira* (T0 vs. T3) and (T3 vs. T6). For the species level (using WMS), there were significant changes comparing T0 vs. T6 in *Blautia_wexlerae*, *Anaerostipes_hadrus*, *Bifidobacterium_longum*, *Bacteroides_stercoris*, *Phocaeicola_vulgatus*, *Anaerobutyricum_hallii* and *Barnesiella_intestinihominis*.

### 3.4. Differences in Alpha Diversity Indices During Synbiotic Supplementation

**Figure 4:**
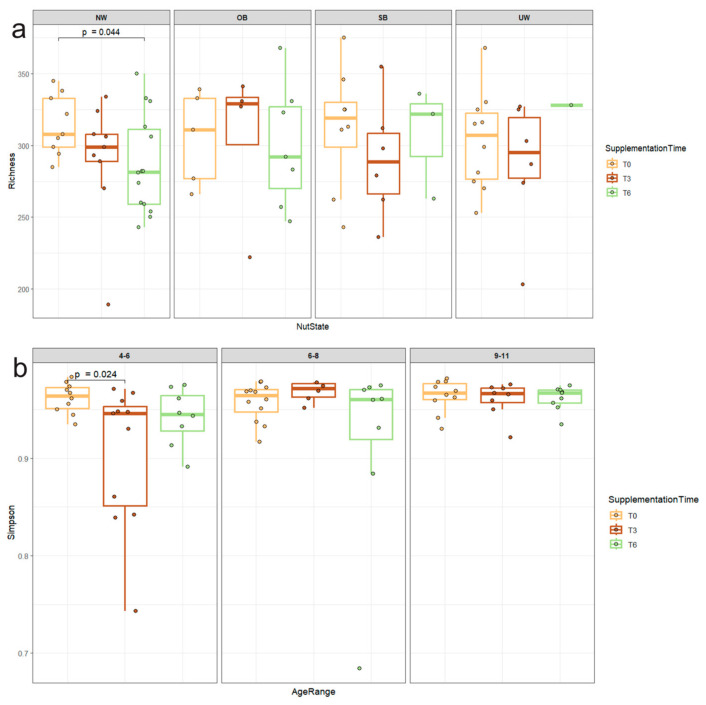
Differences in alpha diversity indices: (**a**) Richness and (**b**) Simpson. The box plot figures show the alpha diversity of the bacterial communities in nutrition status and age according to supplementation time.

The alpha diversity indices (Richness, Shannon, and Simpson) of the gut microbiota of patients with autism were measured in relation to covariates, such as sex, age range, nutritional status, and ASD severity, during the follow-up period for synbiotic supplementation (T0, T3, and T6). The Richness index showed significant differences (*p* < 0.044) when comparing the nutritional status of patients in T0 and T6 of supplementation, and the Simpson index showed a significant difference (*p* < 0.024) when comparing the age range of 4–6 years in T0 vs. T3 of supplementation. There were no significant differences in the Shannon index when sex, age range, nutritional status, and ASD severity were compared with the time of supplementation (Figure 4).

### 3.5. Changes in Beta Diversity During Synbiotic Supplementation

The beta diversity index among the covariates (sex, age, nutritional status and ASD severity) significantly influenced changes in the gut bacterial communities of patients with ASD during the 6-month follow-up period of synbiotic supplementation (Table 4).

### 3.6. Correlation of Bacterial Genera with Synbiotic Time Supplementation and Other Covariates

**Figure 5:**
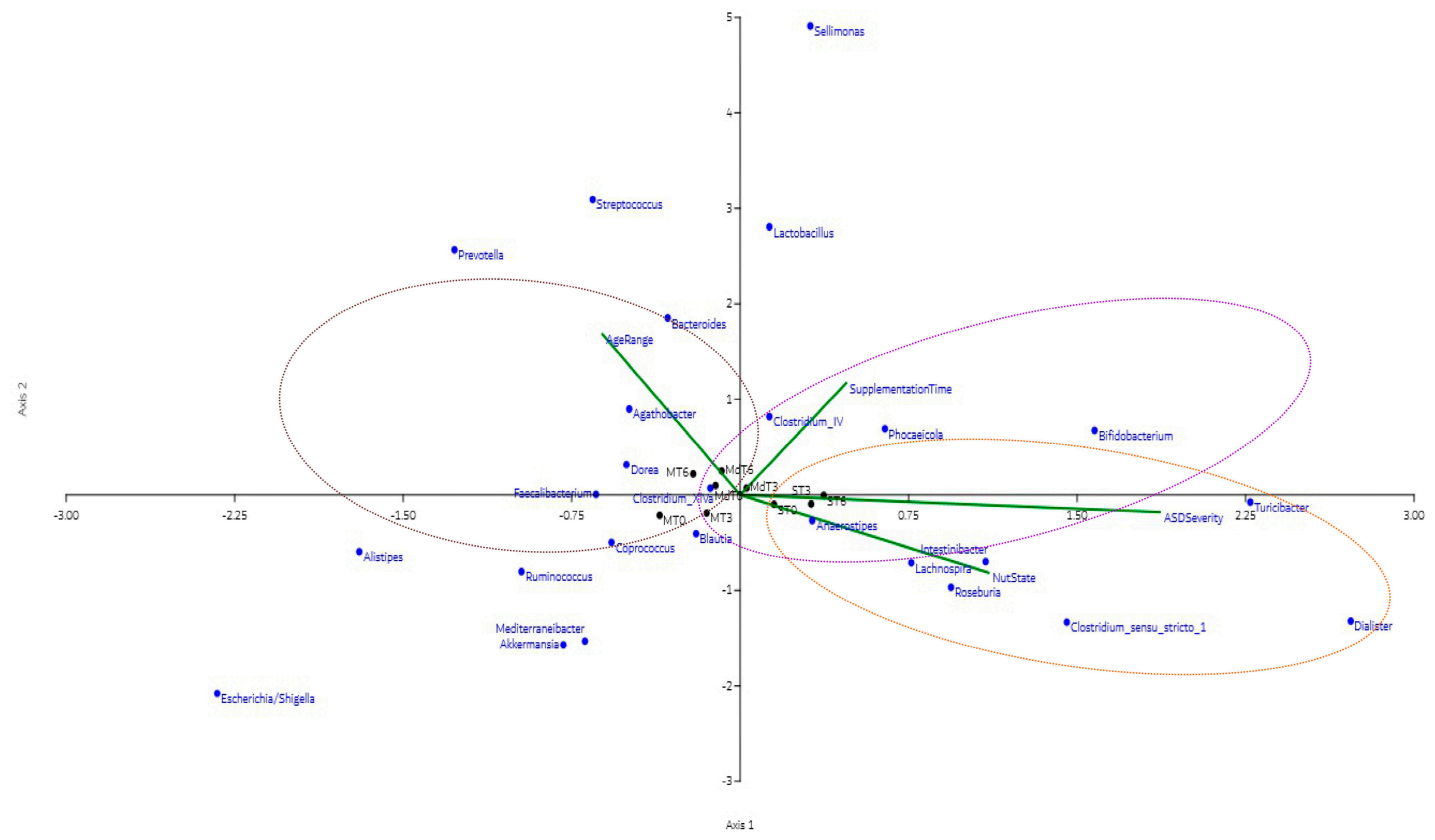
Canonical correspondence analysis (CCA) of bacterial genera according to synbiotic supplementation time and the clinical covariates age, nutritional status, and ASD severity. The first two CCA axes accounted for 87.72% of the constrained variation. The purple ellipse highlights bacterial genera associated with synbiotic supplementation time and the moderate and severe ASD sampling groups; the orange ellipse highlights genera associated with ASD severity and nutritional status; and the brown ellipse highlights genera associated with age. Ellipses are included as visual guides to emphasize these associations and do not represent confidence regions. Thick green arrows represent the explanatory variables, with arrow direction indicating the direction of the association and arrow length reflecting its relative strength. Black points and labels represent the study groups defined by ASD severity and sampling time (MildT0, MildT3, MildT6; ModerateT0, ModerateT3, ModerateT6; and SevereT0, SevereT3, SevereT6), whereas blue points represent bacterial genera.

Canonical correspondence analysis (CCA) at the bacterial genus level between the different supplementation times and the covariates of age, nutritional status, and ASD severity allowed the identification of the correlation between them. The first two CCA axes accounted for 87.72% of the constrained variation in the canonical correspondence analysis (Figure 5). The bacterial genera *Clostridium IV*, *Phocaeicola*, and *Bifidobacterium* are correlated with the time of synbiotic supplementation in moderate and severe ASD cases (purple ellipse); the genera *Anaerostipes*, *Lachnospira*, *Roseburia*, *Intestinibacter*, *Turicibacter*, *Clostridium_sensu_stricto_1* and *Dialister* are correlated to the clinical covariates of ASD severity and nutritional status (orange ellipse), and the bacterial genera *Faecalibacterium*, *Clostridium_XIVa*, *Dorea*, *Agathobacter* and *Bacteroides* are correlated with the age of the patients (brown ellipse).

### 3.7. Correlation of Metabolic Bacterial Pathways and Clinical Variables of Patients with ASD During Synbiotic Supplementation Follow-Up

**Figure 6:**
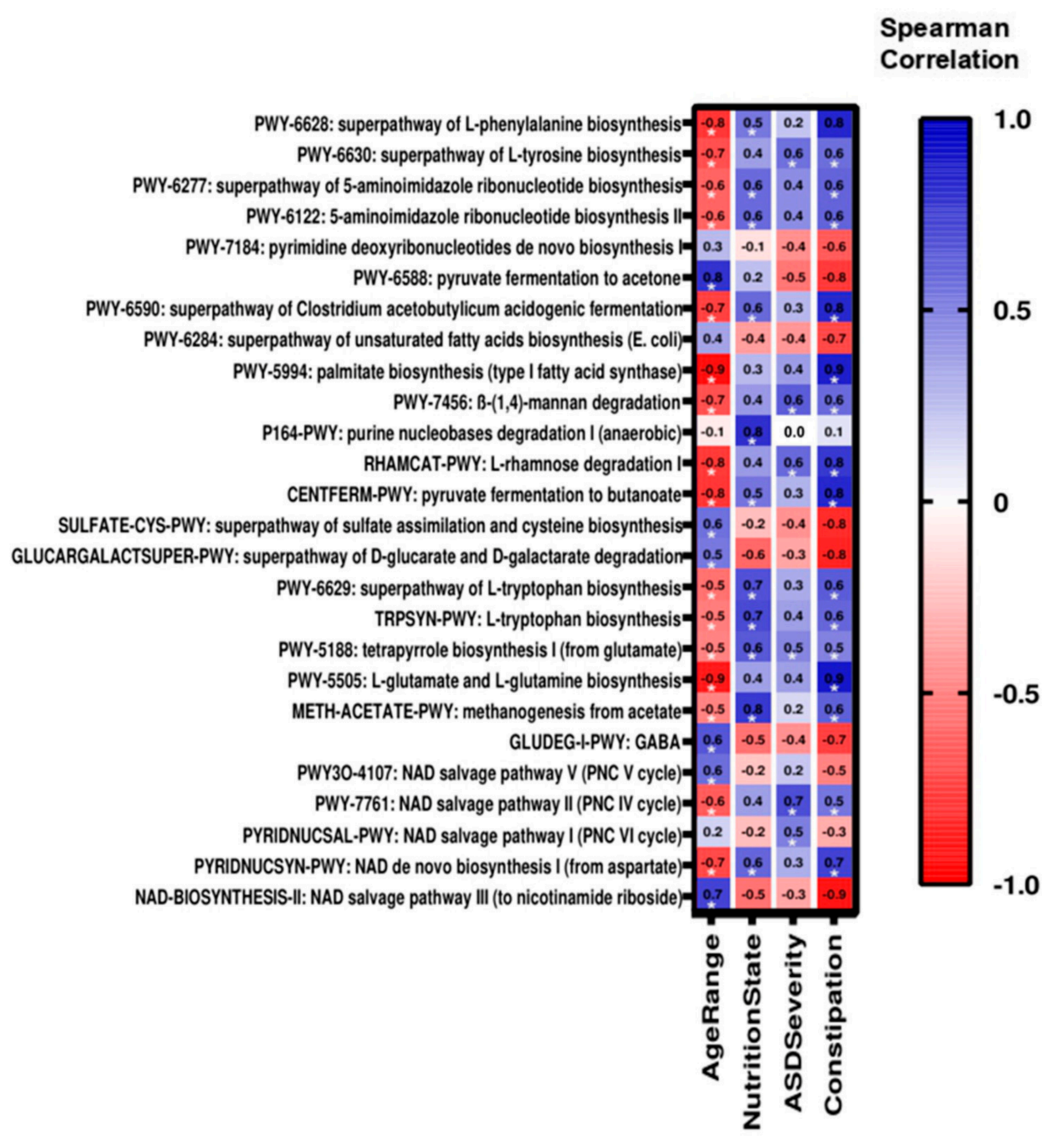
Spearman’s rank correlation between bacterial metabolic pathways and patient evaluation covariates in individuals with ASD during synbiotic supplementation follow-up (T0 = before; T6 = after). * Indicates significance at *p* < 0.05. The Spearman correlation coefficient (r) is within the boxes.

The functional microbiome analysis identified that the abundance of metabolic pathways was correlated with the clinical variables ASD severity, constipation, age and nutritional status of patients with ASD (Figure 6).

The Spearman correlation analysis showed significant positive correlations (*p* < 0.05) between the ASD severity and the metabolic pathways: the superpathway of L-tyrosine biosynthesis, β-(1,4)-mannan degradation, L-rhamnose degradation I, tetrapyrrole biosynthesis I (from glutamate), NAD salvage pathway II (PNC IV cycle), and NAD salvage pathway I (PNC VI cycle). Also, significant positive correlations were found between the nutritional status and metabolic pathways: the superpathway of L-phenylalanine biosynthesis, the superpathway of 5-aminoimidazole ribonucleotide biosynthesis, 5-aminoimidazole ribonucleotide biosynthesis II, the superpathway of *Clostridium acetobutylicum* acidogenic fermentation, purine nucleobases degradation I (anaerobic), pyruvate fermentation to butanoate, the superpathway of L-tryptophan biosynthesis, L-tryptophan biosynthesis, tetrapyrrole biosynthesis I (from glutamate), methanogenesis from acetate and NAD de novo biosynthesis I (from aspartate) (Figure 6).

The same analysis identified a significant positive correlation between the presence of constipation and the metabolic pathways: the superpathway of L-tyrosine biosynthesis, the superpathway of 5-aminoimidazole ribonucleotide biosynthesis, 5-aminoimidazole ribonucleotide biosynthesis II, the superpathway of *Clostridium acetobutylicum* acidogenic fermentation, palmitate biosynthesis (type I fatty acid synthase), β-(1,4)-mannan degradation, L-rhamnose degradation I, pyruvate fermentation to butanoate, the superpathway of L-tryptophan biosynthesis, L-tryptophan biosynthesis, tetrapyrrole biosynthesis I (from glutamate), L-glutamate and L-glutamine biosynthesis, methanogenesis from acetate, NAD salvage pathway II (PNC IV cycle) and NAD de novo biosynthesis I (from aspartate) (Figure 6).

Finally, other correlations were identified by this analysis for the covariate age, such as positive correlations with the metabolic pathways: pyruvate fermentation to acetone, the superpathway of sulfate assimilation and cysteine biosynthesis, the superpathway of D-glucarate and D-galactarate degradation, GABA, NAD salvage pathway V (PNC V cycle), NAD salvage pathway III (to nicotinamide riboside), and negative correlations with the metabolic pathways: the superpathway of L-phenylalanine biosynthesis, the superpathway of L-tyrosine biosynthesis, the superpathway of 5-aminoimidazole ribonucleotide biosynthesis, 5-aminoimidazole ribonucleotide biosynthesis II, the superpathway of *Clostridium acetobutylicum* acidogenic fermentation, palmitate biosynthesis (type I fatty acid synthase), β-(1,4)-mannan degradation, L-rhamnose degradation I, pyruvate fermentation to butanoate, the superpathway of L-tryptophan biosynthesis, L-tryptophan biosynthesis, tetrapyrrole biosynthesis I (from glutamate), L-glutamate and L-glutamine biosynthesis, methanogenesis from acetate, NAD salvage pathway II (PNC IV cycle) and NAD de novo biosynthesis I (from aspartate) (Figure 6).

### 3.8. Changes in Bacterial Metabolic Profiles After Synbiotic Supplementation in Children with ASD

**Figure 7:**
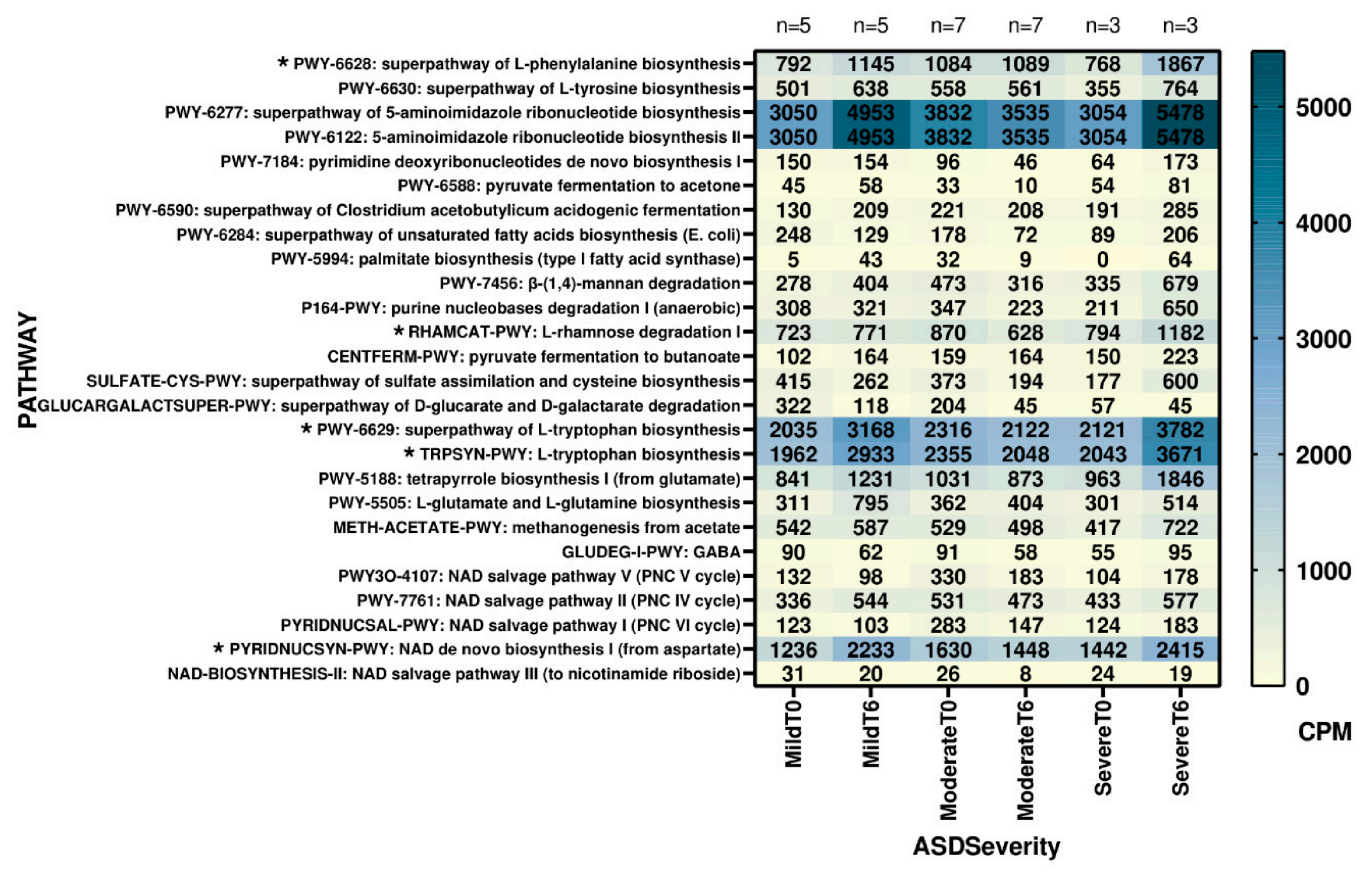
Differences in metabolic pathway abundance in gut microbiota between T0 (before) and T6 (after) according to ASD severity. * *t*-test *p* < 0.05 was considered significant.

Of the 40 metagenomes analyzed, 30 met the predefined coverage criteria based on the UniRef90 database. Twenty-six metabolic pathways potentially relevant to gut–brain axis signaling were selected for further analysis (Figure 7). Pathway abundance differed according to supplementation time and ASD severity.

When comparing abundances, Counts per Million Reads (CPMs) of the pathways between supplementation times, and mild and severe ASD, the superpathway of L-tryptophan biosynthesis and L-tryptophan biosynthesis metabolic pathways were significantly enriched (*p* < 0.05). When comparing the abundances between supplementation times and moderate ASD, the metabolic pathway L-rhamnose degradation I (*p* < 0.03) was statistically significant, and when comparing the abundances between supplementation times and mild ASD, the metabolic pathways NAD de novo biosynthesis I (from aspartate) (*p* < 0.02) and the superpathway of L-phenylalanine biosynthesis (*p* < 0.018) were significant (Figure 7).

## 4. Discussion

Previous studies have suggested an association between gut dysbiosis, gastrointestinal symptoms, and the exacerbation of autistic manifestations in children with ASD [5,24,25]. In the present study, 6 months of multi-strain synbiotic supplementation was associated with significant reductions in gastrointestinal symptoms, as assessed by the Rome IV criteria, and with a decrease in ASD severity according to the total CARS score. These clinical changes were accompanied by significant shifts in gut microbiome composition across multiple taxonomic levels, including phylum, class, order, family, and genus. Similar observations have been reported in previous studies [26], supporting the hypothesis that synbiotic supplementation may contribute to improvements in gastrointestinal and behavioral manifestations in patients with ASD.

The observed changes in CARS scores should be interpreted as reflecting a moderate clinical impact rather than a complete resolution of ASD manifestations. Nevertheless, these improvements are clinically relevant because they involve several functional domains, including emotional response, body use, adaptation to change, auditory response, fear or nervousness, and general impression. Improvements in these areas may translate into better daily functioning, adaptive behavior, and social interaction, and are often noticeable to parents or caregivers in everyday activities. Importantly, the reduction in total CARS score was accompanied by a shift in severity distribution, with fewer children classified in the moderate/severe range after the intervention. These findings suggest that synbiotic-associated clinical changes may have a positive impact on patient functionality and quality of life, although causality cannot be established due to the exploratory design and absence of a placebo-controlled group.

The multi-strain synbiotic formulation was selected to target complementary mechanisms implicated in ASD-related gut dysbiosis rather than a single microbial alteration. The selected probiotic strains have been individually associated with the reinforcement of intestinal barrier integrity, the modulation of immune and inflammatory responses, the production of neuroactive metabolites and short-chain fatty acids, and the restoration of microbial ecological balance. In combination with the prebiotic component, these strains were expected to promote microbiome resilience and functional recovery of the gut ecosystem, thereby supporting gut–brain axis signaling and potentially improving gastrointestinal and behavioral manifestations associated with ASD.

Although the administered probiotic strains did not exhibit significant increases in abundance, marked changes in microbial community structure were observed, suggesting that their biological effects may not depend on numerical dominance within the gut microbiota. Instead, probiotic activity may arise through metabolic, immunological, and ecological interactions within the microbial ecosystem, mediated by bioactive metabolites and signaling molecules that influence host physiology and microbial community function [27,28,29]. Accordingly, the lack of sustained enrichment of the administered taxa suggests that the observed effects were primarily mediated through modulation of the resident microbiota [30].

The observed changes in microbial community structure are consistent with the ecological mechanisms proposed for multi-strain synbiotic formulations, which promote complementary microbial functions involved in restoring microbial ecology and microbiome resilience. Collectively, these probiotic strains have been associated with improved intestinal barrier integrity, the modulation of immune and inflammatory responses, the production of beneficial microbial metabolites, and the regulation of gut–brain axis signaling, all of which are mechanisms implicated in ASD pathophysiology [31,32]. Moreover, the concurrent changes in community structure and microbial metabolic potential support an ecosystem-level mechanism of synbiotic action [33], reinforcing the concept that gut dysbiosis in ASD reflects alterations in microbial function and ecology rather than changes in specific taxa alone.

The biological relevance of these taxonomic changes is supported by the functional roles of the affected microbial groups in gut–brain axis communication. Several of the bacterial genera modulated with synbiotic supplementation, including Bifidobacterium, Anaerostipes, Roseburia, and members of the Lachnospiraceae family, are recognized producers of short-chain fatty acids (SCFAs), particularly butyrate, which promotes intestinal barrier integrity, regulates immune and inflammatory responses, and modulates neuroimmune signaling [34,35]. In addition, the inferred changes in microbial genetic potential-related glutamate/GABA and tryptophan metabolic pathways suggest that synbiotic-induced remodeling of the gut microbiota may influence the production of neuroactive metabolites involved in neurotransmission, synaptic function, and serotonin biosynthesis [36]. Together, these mechanisms provide biological plausibility linking microbiome remodeling with the improvements in gastrointestinal and behavioral manifestations observed in ASD, although causal relationships remain to be established [37].

Regarding changes in abundance following synbiotic supplementation in children with ASD, significant differences were observed at the class (Clostridia) and order (Clostridiales) levels. Notably, members of the genus *Clostridium* showed sustained abundance changes that remained significant throughout the six months of synbiotic supplementation period. A meta-analysis highlighted significant differences in *Clostridium* abundance between ASD and non-ASD children [38]. The spore-forming properties that facilitate the release of pro-inflammatory toxins capable of impacting the central nervous system (CNS) make *Clostridium* a potentially harmful genus that could influence ASD phenotypes [39].

The significant decrease in *Clostridium* after 6 months of supplementation may have benefited the intestinal epithelial barrier by reducing oxidative stress, since high *Clostridium* levels have been associated with low concentrations of benzaldehyde, the reduction of which in the intestine could indicate altered responses to oxidative stress [40]. Intestinal barrier homeostasis is largely due to the oxidative reduction potential conferred by the microbiota, while the CNS modulates oxidative stress levels within the intestine through the vagal cholinergic anti-inflammatory pathway, which may be altered in individuals with ASD due to the dysbiosis they present [41].

In our study, the genus *Sellimonas* stands out, as its abundance remained significantly increased during the 6-month follow-up period of synbiotic supplementation. Before supplementation, there was a significant increase; after three months of supplementation, the increase continued but was not statistically significant; and after six months of supplementation, the increase remained significant. In another study, fecal microbiota transplantation therapy for 12 weeks decreased the abundance of *Sellimonas* [42]. The results of a study on microbiota and sleep in a healthy population suggested a relationship between increased *Sellimonas* levels and increased stage 2 non-rapid eye movement (non-REM) sleep. During this sleep stage, synaptic pruning occurs, allowing the brain to maintain its efficiency and be ready to process new information the following day [43].

Unlike other studies, we compared the severity of ASD with the duration of synbiotic supplementation. Our results showed that supplementation over a 6-month period has significant effects on the diversity of the gut microbiota, and that these effects begin to be identifiable from 3 months of supplementation. The genera *Blautia* (MildT3 vs. MildT6) and *Clostridium XIVa* (ModerateT3 vs. ModerateT6) stand out for their importance in the gut microbiome due to their ability to ferment plant polysaccharides for the production of butyrate, a short-chain fatty acid essential for gut health and anti-inflammatory processes, as well as preventing colonization by pathogenic bacteria [44]. A randomized controlled trial that supplemented children with ASD with probiotics for 3 months found improvements in gastrointestinal and behavioral symptoms as well as observing changes in the severity of autistic symptoms [45].

A study evaluating age-dependent gut dysbiosis in children with ASD found a decrease in beneficial Actinobacteriota (Actinobacteria) and an increase in potentially pathogenic Proteobacteria across all age groups. They also found that the Bacillota (Firmicutes)/Bacteroidota (Bacteroidetes) ratio was decreased, but remained within acceptable limits during middle childhood, suggesting a transient, age-limited rebalancing of the gut microbiota structure [46]. The concurrent suppression of A/P and B/B ratios during the neurodevelopmentally sensitive preschool years reinforces the proposal that early losses of short-chain fatty acid-producing and vitamin-biosynthetic taxa, along with the relative enrichment of endotoxin-producing Proteobacteria, may contribute to the metabolic and immunological disturbances observed in ASD [47].

According to canonical correspondence analysis, in our study, we found that the age covariate is correlated with the bacterial genera *Faecalibacterium*, *Clostridium XIVa*, *Dorea*, *Agathobacter*, and *Bacteroides*. These bacterial genera contribute to gut health through the production of short-chain fatty acids, carbohydrate fermentation, immune system regulation, and the utilization of specific gut nutrients [48]. An exploratory study found that children with ASD, compared to typically developing children, had more pronounced microbiota alterations at younger ages (4–7 years) compared to older ages (8–10 years), suggesting that early interventions targeting gut microbiota modulation may be particularly relevant during early developmental stages [49].

Age-related differences may reflect not only variations in ASD severity but also the intrinsic plasticity and resilience of the developing gut microbiome. Alterations in microbial composition appear to be more pronounced during early childhood [46], when the intestinal ecosystem is still undergoing maturation and is therefore more susceptible to environmental and host-related influences [50]. Conversely, the apparent attenuation of dysbiosis observed with increasing age may partially reflect endogenous mechanisms of ecological resilience or self-repair that promote stabilization of the microbial community over time [30]. However, in children with ASD, these adaptive processes may be constrained by factors such as selective eating behaviors, persistent gastrointestinal symptoms, chronic inflammation, medication use, neurodevelopmental comorbidities, and clinical severity [51]. Therefore, longitudinal controlled studies are needed to determine whether age-related microbiome changes primarily reflect spontaneous ecological recovery, developmental maturation, treatment responsiveness, or a combination of these mechanisms. Furthermore, the greater plasticity of the pediatric microbiome suggests that responses to probiotic or synbiotic interventions may differ according to age, immune maturation, diet, neurodevelopmental stage, and baseline ecological stability [52].

In general, ASD is diagnosed by age 3 in most children with the disorder, although approximately 40% of these children are not first evaluated until age 4 [53]. The gut microbiota has three developmental phases: a developmental phase (3–14 months), a transitional phase (15–30 months), and a stable phase (31–46 months) [54]. It is during this last phase and the transitional phase that patients with ASD typically present with gut microbiota dysbiosis. Therefore, microbiota-directed interventions initiated during early life may have the greatest potential to promote a healthier microbial trajectory, reduce gastrointestinal disturbances, and possibly mitigate some ASD-associated symptoms.

The covariate of supplementation time correlated with the genera *Clostridium IV*, *Phocaeicola*, and *Bifidobacterium*. These bacteria are part of an important group of commensals (beneficial) bacteria in the gut, contributing to the anti-inflammatory effect derived from the production of short-chain fatty acids (SCFAs) [55]. A recent study that evaluated the effects of probiotics on the change in the gut microbiota and brain function of preschool children with ASD concluded that the effect of probiotics did not change significantly with respect to the time variable, suggesting that the observed modifications (changes in brain connectivity) were due to the probiotic supplement (Visbiome^®^) [56]. The covariates of ASD severity and nutritional status were correlated with the genera *Anaerostipes*, *Lachnospira*, *Roseburia*, *Intestinibacter*, *Turicibacter*, *Clostridium sensu stricto 1*, and *Dialister*. Most of these bacterial genera are producers of SCFAs, particularly butyrate, which is important in the control of intestinal anti-inflammatory processes and in the maturation of the immune system. However, changes in the abundance of certain species can lead to inflammatory processes [57].

In the most severe ASD phenotype, higher levels of *Lachnospiraceae* and lower levels of *Faecalibacterium* were found. The abundance of *Lachnospiraceae* has been associated with ASD severity; however, the direction and biological significance of this association remain uncertain [58]. Although members of the *Lachnospiraceae* family are known producers of SCFAs, differences in microbial networks and cross-feeding patterns can result in different physiological effects [59].

The genus *Turicibacter* is correlated with dietary fat and body weight, but its physiological capacity in the host is poorly understood. A study in mice demonstrated that some *Turicibacter* strains decreased serum cholesterol, triglycerides, and adipose tissue mass, identifying them as modulators of host fat biology [60]. Changes in *Turicibacter* abundance have also been associated with metabolic status and inflammatory responses in animal models [61]. In a study characterizing the gut microbiota of Lebanese children, a decrease in the abundance of *Turicibacter* was found in the ASD group compared to the control group, suggesting this genus as a potential biomarker for ASD [62].

In our study, when correlating age with metabolic pathways derived from shotgun analysis, we found a positive correlation between age and GABA metabolic pathway and a negative association with L-glutamate metabolic pathway. Several studies have highlighted the existence of an imbalance in the glutamate and GABAergic systems in relation to ASD [63]. Although GABA alone does not cross the blood–brain barrier, glutamate can be transformed into GABA through the enzymatic action of glutamic acid decarboxylase, which facilitates neurotransmitter transmission and improves communication of the gut–brain axis via the vagus nerve (VN) directly or indirectly, and thus regulating the CNS [64,65]. Children with ASD exhibit alterations in the production of microbial metabolites resulting from gut dysbiosis [11,66]. Glutamate and GABA are directly involved in brain development, as well as synaptogenesis, memory, behavioral regulation, locomotor activity, and gastrointestinal function [67]. Decreased glutamate levels have been associated with the severity of social and behavioral symptoms in ASD [68].

We found negative associations between age of participants and the metabolic pathways associated with the production of amino acids (superpathway of L-phenylalanine biosynthesis, superpathway of L-tyrosine biosynthesis, L-glutamate and L-glutamine biosynthesis) that play a key role in the nervous system. The negative association between age and these biosynthetic pathways suggests that microbial metabolic deficiencies in ASD may vary with age and reflect changes in the regulation of essential amino acids and neuroactive molecules involved in cognitive function, neurotransmitter synthesis, and brain development, leading to an exacerbation of neurological symptoms over time. [69,70]. However, whether these alterations contribute to neurological symptom progression remains to be established.

Excessive glutamate levels can disrupt synaptic pruning and neuronal network formation, and chronic excitotoxicity can compromise neuronal survival and promote neurodevelopmental alterations. Such alterations may underlie the cognitive and behavioral challenges characteristic of ASD [71]. Furthermore, elevated phenylalanine levels can disrupt the metabolic pathways of neurotransmitters such as dopamine, leading to behavioral and emotional abnormalities in individuals with ASD [72]. A study in mice of different ages found age-dependent changes in microbial taxa associated with gut dysbiosis and pro-inflammatory signaling, as well as an age-related decrease in the levels of gut microbe-dependent bioactive metabolites containing tryptophan-derived indoles [73]. Reduced tryptophan levels can lead to insufficient serotonin synthesis, an important neurotransmitter for regulating mood and behavior [74]. Abnormalities in serotonin function are closely associated with anxiety, depression, and social impairments commonly observed in individuals with ASD [75].

A study evaluating the metabolism of relevant amino acids in ASD found a negative correlation between age and ASD severity. This association suggests that older children tend to present with less severe ASD symptoms compared to younger children [70]. Abnormalities in amino acid metabolism are especially relevant to ASD pathology due to their crucial role in neurotransmitter synthesis, synaptic function, and overall neurological development [76]. Current evidence supports an association between gut dysbiosis and ASD-related clinical features and suggests that the microbiome may represent a potentially modifiable therapeutic target [77]. Several studies have demonstrated that symbiotic supplementation significantly improves behavioral symptoms in ASD [78].

The correlation between nutritional status and metabolic pathways revealed several metabolic pathways associated with inflammatory processes (superpathway of *Clostridium acetobutylicum* acidogenic fermentation) and metabolic diseases (superpathway of L-phenylalanine biosynthesis, superpathway of 5-aminoimidazole ribonucleotide biosynthesis and 5-aminoimidazole ribonucleotide biosynthesis II). Children with ASD often have a nutritional status tending towards overweight and obesity, due to food selectivity, low physical activity, and high consumption of processed foods high in fat and sugar [79]. Obesity may increase long-term cardiometabolic risk in children with ASD [80]. On the other hand, a positive correlation was also found between nutritional status and metabolic pathways associated with anti-inflammatory processes (superpathway of L-tryptophan biosynthesis, L-tryptophan biosynthesis and tetrapyrrole biosynthesis I from glutamate). Synbiotic therapy, aimed at modulating the gut microbiota and the gut–brain axis [81], has demonstrated a significant improvement in symptoms associated with obesity and neurobehavioral disorders [82].

When constipation was correlated with different metabolic pathways, we found that constipation is positively related to the tryptophan metabolism pathway (the superpathway of L-tryptophan biosynthesis and L-tryptophan biosynthesis). Studies in murine models of ASD reported a 50% reduction in the neurotransmitter 5-hydroxytryptamine (serotonin production pathway) in the gut, suggesting that luminal tryptophan may be metabolized by the gut microbiota, further limiting its availability to the host. This reduction is related to intestinal transit time and the *Blautia* bacterial genus abundance [83,84]. Moreover, significant changes in tryptophan metabolic pathways (superpathway of L-tryptophan biosynthesis and L-tryptophan biosynthesis) were found in mild (T0 vs. T6) and severe (T0 vs. T6) levels. These findings are consistent with the hypothesis that tryptophan-derived microbial metabolites may influence brain function through the gut–brain axis and could contribute to behavioral manifestations in ASD [85]. Therefore, modulation of the gut microbiota could play a crucial role in determining tryptophan availability to the host, balancing microbial tryptophan metabolism and, consequently, the serotonin production and tryptophan degradation pathways [86].

Regarding the changes observed in metabolic pathways during the synbiotic supplementation, a significant decrease in the rhamnose metabolic pathway (L-rhamnose degradation I) was found in patients with moderate ASD severity (T0 vs. T6). In Gram-negative bacteria, rhamnose is found in lipopolysaccharide (LPS) [87]. This LPS triggers robust immune responses and may play a role in neuroinflammatory pathways that can be dysregulated in ASD, leading to the heterogeneity of ASD phenotypes [88]. The metabolic pathways of NAD de novo biosynthesis I from aspartate and the superpathway of L-phenylalanine biosynthesis, related to tryptophan metabolism [89], were found to be enriched in mild ASD (T0 vs. T6). Although elevated phenylalanine levels have been associated with altered dopamine metabolism, the enrichment of tryptophan-related pathways may influence serotonin biosynthesis through the gut–brain axis. However, whether these metabolic changes contribute to improvements in ASD behavioral symptoms remains to be established [70].

Several experimental and clinical studies suggest that probiotics and synbiotics may modulate neurotransmitter-related pathways involved in gut–brain axis signaling, including restoration of serotonin homeostasis in the peripheral and central nervous system, which are dysregulated in ASD [81,90]. Although these mechanisms provide biological plausibility for the reported clinical benefits, the molecular pathways underlying these effects remain incompletely understood, and much of the mechanistic evidence derives from preclinical studies.

### Strengths and Limitations

A major strength of this study is the integrated characterization of the gut microbiome by evaluating both taxonomic composition and metabolic functions and relating these findings to clinically relevant covariates, including sex, age, nutritional status, ASD severity, and constipation. This comprehensive approach provided a broader understanding of microbiome alterations associated with ASD and their response to synbiotic supplementation. Future studies should incorporate functional validation and larger stratified cohorts to further elucidate the effects of synbiotics across different ASD phenotypes.

The main limitations of this study include the relatively small sample size, restricted fecal sampling, the absence of a placebo-controlled group, and the lack of post-intervention follow-up, which precluded evaluation of the persistence of microbiome and clinical changes after discontinuation of synbiotic supplementation. The small sample size also limits the statistical power of this exploratory longitudinal study and precludes definitive conclusions regarding efficacy or causality. Therefore, the observed clinical and microbiome-related changes should be interpreted as preliminary findings that may inform effect size estimation and sample size calculations for future adequately powered placebo-controlled randomized trials. Another potential limitation is the confounding effect of concurrent behavioral therapy. Although none of the participants received pharmacological treatment before or during the intervention, seven children received behavioral therapy during the study period, which may have contributed to improvements in functional and social domains assessed by CARS. Future studies should systematically control for concurrent educational, behavioral, occupational, and pharmacological interventions. Additionally, stratifying patients according to ASD phenotypic complexity—including epilepsy, medication use, sleep disorders, and other neurodevelopmental comorbidities—may help to better understand variability in microbiome composition and intervention response. Although dietary intake was prospectively monitored throughout the same clinical trial and reported separately [79], with no significant changes in nutritional status or macro- and micronutrient intake during follow-up, residual dietary effects on the gut microbiome cannot be completely excluded. Finally, although metagenomic analyses provide insights into functional pathways, experimental validation is still required to confirm their biological relevance and microbial contributions to host physiology.

## 5. Conclusions

In this longitudinal study, six months of multi-strain synbiotic supplementation were associated with reductions in gastrointestinal symptoms and overall ASD severity, as reflected by lower total CARS scores and changes in several CARS domains. These clinical changes were accompanied by measurable shifts in the gut microbiome across multiple taxonomic levels, including changes within *Clostridiales* and in key families (e.g., *Lachnospiraceae*, *Veillonellaceae*, *Clostridiaceae_1*, and *Akkermansiaceae*) and genera such as *Blautia*, *Mediterraneibacter*, *Anaerostipes*, *Akkermansia*, *Clostridium XIVa*, and *Sellimonas*. Functional metagenomic profiling further supported these observations by revealing changes in microbial metabolic potential, including enrichment of tryptophan biosynthesis pathways and a reduction in L-rhamnose degradation.

Taken together, the concordance between clinical improvements and microbiome taxonomic and functional remodeling supports the hypothesis that synbiotics may influence pathways relevant to the microbiota–gut–brain axis in children with ASD. However, the persistence of these effects following treatment discontinuation and their variability according to age, clinical complexity, and microbiome resilience remain to be determined in controlled longitudinal studies. Interpretation of these findings should be considered in light of the study’s limitations, including the modest sample size, the lack of a parallel control group, the absence of a post-intervention follow-up period and the fact that metagenomic analyses were conducted in only a subset of samples. Future adequately powered, randomized controlled trials are needed to confirm causality, identify predictors of response, and define optimal dosing and duration of synbiotic interventions.

## Figures and Tables

**Table 1:** Characteristics of patients with autism spectrum disorder.

	T0 (Before) n = 25	T6 (After) n = 25
Characteristic	Mean ± SD, (%)	Mean ± SD, (%)
Age (years)	6.72 ± 2.03	7.48 ± 2.02
Sex		
Female	4 (16.0)	4 (16.0)
Male	21 (84.0)	21 (84.0)
Nutrition State		
Underweight	6 (24.0)	1 (4.0)
Normal weight	**9 (36.0)**	**14 (56.0)**
Overweight	6 (24.0)	3 (12.0)
Obesity	4 (16.0)	7 (28.0)
ASD Severity **^a^**		
Mild	8 (32.0)	**16 (64.0)**
Moderate	7 (28.0)	9 (36.0)
Severe	**10 (40.0)**	0 (0.0)
Gastrointestinal Symptoms **^b^**		
Constipation	8 (32.0)	1 (4.0) *
Irritable bowel syndrome	**12 (48.0)**	4 (16.0) *
Reflux	10 (40.0)	0 (0.0) *
Vomiting	9 (36.0)	0 (0.0) *
Abdominal Pain	9 (36.0)	3 (12.0) *
Flatulence	**19 (76.0)**	9 (36.0) *

Values are expressed as mean and standard deviation. For proportions and percentages, values are shown in parentheses. The higher percentage value is highlighted in bold. Z-scores were calculated using AnthroPlus v1.0.4 software according to sex- and age-specific reference values [19]. ^a^ ASD severity was assessed using the CARS, and ^b^ gastrointestinal symptoms were evaluated according to the Rome IV criteria [23]. * *p*-values < 0.05 from Fisher’s exact test were considered statistically significant.

**Table 2:** CARS score for the ASD group T0 (before) and T6 (after) synbiotic supplementation.

Variable	Mean		*p*-Value	95% CI ^†^
	T0	T6		
Relation to people	2.00 ± 0.56	1.90 ± 0.41	0.409	−0.14–0.34
Imitation	2.14 ± 0.81	1.88 ± 0.51	0.102	−0.05–0.57
Emotional response	2.72 ± 0.87	1.94 ± 0.65	**0.001**	0.35–1.2
Body use	2.52 ± 0.82	1.86 ± 0.45	**0.001**	0.29–1.0
Object use	2.20 ± 0.69	1.86 ± 0.40	**0.021**	0.05–0.62
Adaptation to change	2.70 ± 0.74	2.14 ± 0.49	**0.004**	0.20–0.91
Visual response	1.98 ± 0.80	1.68 ± 0.43	0.113	−0.07–0.67
Listening response	2.38 ± 0.75	1.96 ± 0.32	**0.019**	0.07–0.76
Taste, smell, touch	2.12 ± 0.77	1.86 ± 0.42	0.097	−0.05–0.57
Fear or nervousness	2.54 ± 0.89	2.02 ± 0.42	**0.022**	0.08–0.95
Verbal communication	2.68 ± 0.88	2.46 ± 0.72	0.235	−0.15–0.59
Nonverbal communication	1.84 ± 0.51	1.74 ± 0.54	0.346	−0.11–0.31
Activity level	2.02 ± 0.88	1.72 ± 0.54	0.118	−0.08–0.68
Intellectual response	1.92 ± 0.75	1.70 ± 0.52	0.156	−0.09–0.53
General impression	2.50 ± 0.56	2.18 ± 0.56	**0.010**	0.08–0.55
Total score *	33.84 ± 5.95	28.06 ± 0.46	**0.007**	1.7–9.8

Values are expressed as mean ± standard deviation (SD). Between-group comparisons were performed using the Student’s *t*-test, and *p* values < 0.05 were considered statistically significant. Statistically significant values are shown in bold. * The total score corresponds to the sum of items I–XV. † CI, confidence interval; values are presented as 95% CIs.

**Table 3:** Comparative analysis of taxon abundance among groups compared with synbiotic supplementation time.

Taxonomic Rank	Taxa	Abundance Taxonomic (%) Mean and ±SD			*p*-Value Between Groups (*t*-Test)			95% CI		
		T0 n = 25	T3 n = 25	T6 n = 25	T0 vs. T3 n = 25	T3 vs. T6 n = 25	T0 vs. T6 n = 25	T0 vs. T3 n = 25	T3 vs. T6 n = 25	T0 vs. T6 n = 25
Phylum	Bacillota	77.20 ± 9.03	72.19 ± 20.29	69.62 ± 13.37	↓0.262	↓0.588	↓0.015	−3.98–14.01	−7.06–12.19	1.64–13.51
	Actinobacteria	9.30 ± 6.50	13.25 ± 13.60	13.10 ± 11.74	↑0.113	↓0.960	↑0.064	−8.88–0.99	−5.73–6.02	−7.84–0.24
	Bacteroidetes	10.13 ± 5.32	8.93 ± 9.23	15.03 ± 12.89	↓0.584	↑0.062	↑0.094	−3.26–5.67	−12.54–0.33	−10.71–0.90
	Proteobacteria	2.37 ± 3.58	3.09 ± 9.61	1.78 ± 2.40	↑0.742	↓0.525	↓0.467	−5.11–3.69	−2.87–5.48	−1.06–2.25
	Verrucomicrobia	0.77 ± 1.60	2.44 ± 4.68	0.39 ± 0.74	↑0.062	↓0.033	↓0.240	−3.44–0.08	0.18–3.9	−0.26–1.01
Class	*Clostridia*	72.55 ± 9.64	67.07 ± 20.28	63.35 ± 13.49	↓0.239	↓0.435	↓0.005	−3.87–14.83	−5.96–13.41	3.14–15.26
	*Bacteroidia*	10.21 ± 5.49	9.06 ± 9.24	15.03 ± 12.89	↓0.604	↑0.067	↑0.101	−3.36–5.66	−12.39–0.44	−10.66–1.01
	*Actinobacteria*	7.33 ± 6.55	10.73 ± 12.82	10.79 ± 10.87	↑0.141	↑0.983	↑0.084	−8.01–1.21	−5.85–5.73	−7.41–0.49
	*Negativicutes*	2.18 ± 3.53	1.50 ± 1.85	2.90 ± 4.66	↓0.132	↑0.071	↑0.187	−0.21–1.58	−2.93–0.12	−1.81–0.37
	*Gammaproteobacteria*	1.73 ± 3.38	2.88 ± 9.63	1.13 ± 2.29	↑0.592	↓0.397	↓0.440	−5.49–3.20	−2.42–5.91	−0.97–2.17
	*Bacilli*	1.05 ± 1.14	1.65 ± 3.62	1.57 ± 2.13	↑0.431	↓0.929	↑0.299	−2.14–0.97	−1.73–1.88	−1.53–0.49
	*Verrucomicrobiae*	0.76 ± 1.60	2.44 ± 4.68	0.39 ± 0.74	↑0.062	↓0.033	↓0.241	−3.44–0.08	0.18–3.92	−0.26–1.01
	*Erysipelotrichia*	0.77 ± 0.55	1.26 ± 1.55	0.77–0.72	↑0.100	↓0.078	↓0.965	−1.08–0.10	−0.06–1.05	−0.25–0.26
	*Betaproteobacteria*	0.20 ± 0.31	0.12 ± 0.16	0.37 ± 0.36	↓0.208	↑0.001	↑0.050	−0.04–0.21	−0.39-(-)0.10	−0.34-(-)0.00
Order	*Clostridiales*	72.55 ± 9.64	67.08 ± 20.29	63.35 ± 13.49	↓0.239	↓0.435	↓0.005	−3.88–14.82	−5.96–13.42	3.14–15.26
	*Bacteroidales*	10.21 ± 5.49	8.93 ± 9.23	15.03 ± 12.89	↓0.564	↑0.062	↑0.101	−3.23–5.79	−12.54–0.33	−10.66–1.01
	*Bifidobacteriales*	7.31 ± 6.55	10.76 ± 12.78	10.74 ± 10.90	↑0.135	↓0.996	↑0.086	−8.05–1.15	−5.77–5.80	−7.39–0.52
	*Enterobacteriales*	1.69 ± 3.36	2.74 ± 9.66	1.09 ± 2.29	↑0.623	↓0.423	↓0.436	−5.41–3.30	−2.53–5.83	−0.96–2.16
	*Veillonellales*	1.43 ± 3.50	1.02 ± 1.82	2.20 ± 4.45	↓0.317	↑0.083	↑0.045	−0.041–1.22	−2.52–0.16	−1.53-(-)0.01
	*Lactobacillales*	1.01 ± 1.14	1.64 ± 3.61	1.54 ± 2.13	↑0.407	↓0.905	↑0.293	−2.18–0.91	−1.70–1.91	−1.53–0.48
	*Verrucomicrobiales*	0.76 ± 1.60	2.44 ± 4.68	0.39 ± 0.74	↑0.061	↓0.033	↓0.241	−3.44-0.08	0.18–3.92	−0.26–1.01
	*Burkholderiales*	0.20 ± 0.31	0.12 ± 0.15	0.37 ± 0.36	↓0.220	↑0.002	↑0.050	−0.05–0.21	−0.39-(-)0.10	−0.34-(-)0.00
	*Selenomonadales*	0.03 ± 0.17	0.05 ± 0.23	0.07 ± 0.19	↑0.149	↑0.712	↑0.427	−0.04–0.00	−0.12–0.08	−0.13–0.06
Family	*Lachnospiraceae*	37.43 ± 11.66	37.72 ± 13.18	30.07 ± 9.74	↑0.921	↓0.027	↓0.021	−6.25–5.68	0.94 ± 14.36	1.19–13.53
	*Ruminococcaceae*	26.96 ± 8.89	22.69 ± 10.97	26.41 ± 10.04	↓0.136	↑0.156	↑0.801	−1.44–9.99	−8.97–1.52	−3.86–4.95
	*Bifidobacteriaceae*	7.31 ± 6.55	10.76 ± 12.78	10.74 ± 10.90	↑0.135	↑0.996	↑0.086	−8.05–1.15	−5.77–5.80	−7.39–0.52
	*Bacteroidaceae*	4.94 ± 3.32	5.78 ± 7.89	6.62 ± 7.61	↑0.649	↑0.692	↑0.346	−4.59–2.91	−5.20–3.51	−5.30–1.93
	*Prevotellaceae*	3.10 ± 5.14	1.34 ± 2.81	4.44 ± 8.05	↓0.074	↑0.075	↑0.436	−0.18–3.69	−6.53–0.33	−4.85–2.16
	*Clostridiaceae_1*	3.26 ± 3.75	2.52 ± 3.88	1.76 ± 2.03	↓0.352	↓0.283	↓0.010	−0.87–2.35	−0.66–2.17	0.39–2.60
	*Veillonellaceae*	1.43 ± 3.50	1.02 ± 1.82	2.20 ± 4.45	↓0.317	↑0.083	↑0.045	−0.41–1.22	−2.52–0.16	−1.53-(-)0.01
	*Akkermansiaceae*	0.76 ± 1.60	2.44 ± 4.68	0.39 ± 0.74	↑0.061	↓0.033	↓0.241	−3.44–0.08	0.18–3.9	−0.26–1.01
	*Streptococcaceae*	0.80 ± 1.09	1.32 ± 3.62	0.97 ± 1.72	↑0.498	↓0.675	↑0.687	−2.06–1.03	−1.34–2.03	1.02–0.68
	*Lactobacillaceae*	0.08 ± 0.13	0.17 ± 0.43	0.19 ± 0.42	↑0.262	↑0.694	↑0.179	−0.23–0.06	−0.16–0.11	−0.27–0.05
Genus	*Faecalibacterium*	14.37 ± 8.04	12.08 ± 8.91	16.19 ± 10.90	↓0.271	↑0.086	↑0.333	−1.91–6.49	−8.85–0.63	−5.61–1.98
	*Blautia*	12.85 ± 7.42	15.33 ± 7.97	10.50 ± 5.09	↑0.184	↓0.011	↑0.141	−6.22–1.26	−0.83–5.53	1.19–8.47
	*Bifidobacterium*	7.31 ± 6.55	10.76 ± 12.78	10.74 ± 10.90	↑0.135	↓0.996	↑0.086	−8.05–1.15	−5.77–5.8	−7.39–0.52
	*Prevotella*	2.90 ± 5.12	1.02 ± 2.71	4.01 ± 8.07	↓0.053	↑0.084	↑0.522	−0.02–3.77	−6.40–0.43	−4.63–2.41
	*Mediterraneibacter*	2.36 ± 1.68	1.77 ± 1.38	1.51 ± 1.25	↓0.090	↓0.479	**↓0.047**	−0.09–1.29	−0.47–0.99	0.01–1.69
	*Anaerostipes*	2.33 ± 2.34	2.05 ± 1.73	1.34 ± 0.97	↓0.600	**↓0.048**	↓0.052	−0.80–1.36	0.00–1.40	−0.01–1.98
	*Dorea*	1.24 ± 0.56	1.44 ± 1.12	1.01 ± 0.78	↑0.443	↓0.116	↓0.215	−0.73–0.33	−0.11–0.96	−0.13–0.58
	*Akkermansia*	0.76 ± 1.60	2.44 ± 4.68	0.39 ± 0.74	↑0.061	**↓0.033**	↑0.241	−3.44–0.086	0.18–3.9	−0.26–1.01
	*Streptococcus*	0.69 ± 1.08	1.26 ± 3.52	0.95 ± 1.72	↑0.442	↓0.705	↑0.518	−2.08–0.94	−1.35–1.97	1.09–0.56
	*Clostridium_XlVa*	0.43 ± 0.28	0.34 ± 0.23	0.55 ± 0.44	↓0.231	**↓0.027**	↓0.128	−0.05–0.22	−0.39-(-)0.02	−0.28–0.03
	*Sellimonas*	0.01 ± 0.04	0.04 ± 0.08	0.08 ± 0.18	**↑0.030**	↑0.194	**↑0.041**	−0.04–0.00	−0.10–0.02	−0.12–0.00
	*Megamonas*	0.0025 ± 0.004	0.0094 ± 0.038	0.0666 ± 0.196	↑0.374	↑0.123	↑0.115	−0.02–0.00	−0.13–0.01	−0.14–0.01
	*Lactobacillus*	0.03 ± 0.09	0.02 ± 0.03	0.06–0.17	↓0.383	↑0.222	↑0.274	−0.02–0.06	−0.11–0.02	−0.07–0.02
Species	*Faecalibacterium_prausnitzii*	7.20 ± 4.60	-	7.94 ± 6.21	-	-	↑0.584	-	-	−4.24–2.76
	*Blautia_wexlerae*	4.48 ± 3.4	-	2.7 ± 1.94	-	-	↓**0.038**	-	-	−0.087–3.48
	*Anaerostipes_hadrus*	2.39 ± 2.95	-	1.42 ± 1.12	-	-	↓**0.049**	-	-	−0.46–2.40
	*Bifidobacterium_longum*	1.97 ± 2.46	-	4.02 ± 5.22	-	-	↑**0.022**	-	-	−4.67–0.55
	*Bacteroides_stercoris*	1.37 ± 2.49	-	2.39 ± 4.87	-	-	↑**0.025**	-	-	−3.49–1.45
	*Phocaeicola_vulgatus*	1.36 ± 1.19	-	2.68 ± 4.62	-	-	↑**0.013**	-	-	−3.48–0.84
	*Blautia_obeum*	1.06 ± 0.94	-	0.70 ± 0.76	-	-	↓0.369	-	-	−0.19–0.90
	*Anaerobutyricum_hallii*	0.93 ± 0.92	-	0.61 ± 0.51	-	-	↓**0.028**	-	-	−0.16–0.79
	*Candidatus_Cibionibacter_quicibialis*	0.92 ± 1.21	-	0.78 ± 1.16	-	-	↓0.699	-	-	−0.61–0.91
	*Barnesiella_intestinihominis*	0.67 ± 0.93	-	0.49 ± 0.53	-	-	↓**0.044**	-	-	−0.30–0.66

Where T0 = baseline, T3 = 3 months and T6 = 6 months of treatment. Significant differences set at *p* < 0.05 are highlighted in bold. Phylum to genus levels were analyzed with 16S rRNA V3–V4 region, and at the species level, they were analyzed with WMS. ↑ indicates an increase, whereas ↓ indicates a decrease between sampling time points.

**Table 4:** Influence of sex, age, nutritional status and ASD severity on differences in gut microbiome bacterial communities of patients with ASD during supplementation follow-up.

Covariate	Degrees of Freedom	Bray–Curtis: F Value (*p*-Value)
Sex	1	1.89 (0.008 *)
Age	1	2.10 (0.001 *)
Nutritional Status	3	1.42 (0.004 *)
ASD Severity	2	1.83 (0.001 *)

ADONIS2 test, Bray–Curtis dissimilarity * indicates significance at 0.05 level.

## Data Availability

All 16S rDNA and Whole Metagenomic Sequence files and corresponding mapping files for the samples utilized in this study have been deposited in the NCBI BioSample repository. Interested parties may access these resources using the provided Accession Number PRJNA1425957, which can be found at the following link: https://www.ncbi.nlm.nih.gov/bioproject/?term=PRJNA1425957 (accessed on 27 May, 2026).

## Abbreviations

The following abbreviations are used in this manuscript:

ASD	Autism Spectrum Disorder
NT	Neurotypical
CARS	Childhood Autism Rating Scale
INP	Instituto Nacional de Pediatría
CRIDI-ASD	Autism Spectrum Disorder Diagnostic Interview
CFU	Colony-Forming Unit
WHO	World Health Organization
BMI	Body Mass Index
WMS	Whole Metagenomic Sequencing
bp	Base Pair
NCBI	National Center for Biotechnology Information
ASV	Amplicon Sequence Variant
PCoA	Principal Coordinates Analysis
CCA	Canonical Correspondence Analysis
PAST	Paleontological Statistics
CNS	Central Nervous System
SCFAs	Short-Chain Fatty Acids
GABA	Gamma-Aminobutyric Acid
VN	Vagus Nerve
NS	Nervous System
LPS	Lipopolysaccharide
CPM	Counts per Million Reads
